# Significant Local-Scale Plant-Insect Species Richness Relationship Independent of Abiotic Effects in the Temperate Cape Floristic Region Biodiversity Hotspot

**DOI:** 10.1371/journal.pone.0168033

**Published:** 2017-01-11

**Authors:** Jurene E. Kemp, Allan G. Ellis

**Affiliations:** Botany and Zoology Department, Stellenbosch University, Matieland, South Africa; Lakehead University, CANADA

## Abstract

Globally plant species richness is a significant predictor of insect richness. Whether this is the result of insect diversity responding directly to plant diversity, or both groups responding in similar ways to extrinsic factors, has been much debated. Here we assess this relationship in the Cape Floristic Region (CFR), a biodiversity hotspot. The CFR has higher plant diversity than expected from latitude (i.e., abiotic conditions), but very little is known about the diversity of insects residing in this region. We first quantify diversity relationships at multiple spatial scales for one of the dominant plant families in the CFR, the Restionaceae, and its associated insect herbivore community. Plant and insect diversity are significantly positively correlated at the local scales (10–50 m; 0.1–3 km), but not at the regional scales (15–20 km; 50–70 km). The local scale relationship remains significantly positively correlated even when accounting for the influence of extrinsic variables and other vegetation attributes. This suggests that the diversity of local insect assemblages may be more strongly influenced by plant species richness than by abiotic variables. Further, vegetation age and plant structural complexity also influenced insect richness. The ratio of insect species per plant species in the CFR is comparable to other temperate regions around the world, suggesting that the insect diversity of the CFR is high relative to other areas of the globe with similar abiotic conditions, primarily as a result of the unusually high plant diversity in the region.

## Introduction

Arthropods associated with plants constitute a major part of the earth’s biodiversity [1]. Since herbivores feed on plants, it is generally accepted that herbivorous insect diversity should increase with an increase in plant diversity [2–4], and this should subsequently also lead to an increase in predacious insect diversity [5]. However, both the strength of the association between these groups and the key determinants of the relationship, are still debated [6]. The strength and slope of this relationship could vary between regions due to variation in insect host specialisation [7], plant biomass [8], plant phylogenetic diversity [4,9], or abiotic factors [10–12].

Speciation resulting from evolutionary transitions of specialist herbivores from one host to another has been inferred as an important mechanism driving arthropod diversity [13]. If insects are highly specialised on plants, a high diversity of plants should lead to a high diversity of insect herbivores at both the community and regional scales, and thus a positive association between plant and insect richness is expected [2,4,5]. Because insects are often specialised on plants at the generic or family level [14–16], a community of distantly related plant species will more likely fall within the host range of a larger variety of herbivores, resulting in high herbivore diversity in phylogenetically diverse plant communities [4]. Castagneyrol et al. [9] suggest the strength of plant-insect richness relationship versus the strength of insect richness and plant phylogenetic relationships is contingent on the patterns of herbivore specialisation. The relationship between insect richness and plant phylogenetic diversity will be stronger than the alternative if insects are specialised at higher taxonomic levels.

However, the positive correlation between plant and insect diversity is not necessarily the result of a direct association. For example, Hawkins & Porter [10] found that once environmental variables were controlled for, plant host diversity and Californian butterfly diversity were not correlated. Craft et al. [17] showed that while genetic structure of tropical insects is in some cases associated with host specialisation, it often mirrors other landscape gradients. These patterns suggest that the plant-insect diversity relationship might arise due to similar responses of both groups to environmental gradients. In addition, properties of plant communities besides species/phylogenetic diversity may influence insect herbivore diversity, such as vegetation structure [18], or plant phenophase [19].

The majority of studies investigating plant-insect diversity relationships have focused on diverse tropical systems [20–23], while other hyperdiverse systems have received much less attention. The Cape Floristic Region (CFR) of South Africa is a recognised biodiversity hotspot [24] that contains more than 9000 plant species in 90 000 km^2^ [25]. While plant species richness of the major floristic kingdoms can be predicted by environmental factors, the CFR represents a clear exception, containing more than twice the plant species richness per unit area than predicted from environmental conditions [26]. The limited work on CFR insect diversity suggests that Cape plant and insect diversity may be positively correlated [27–30]. Authors, however, disagree on whether CFR insect diversity is high [27,31], depauperate [32,33] or comparable to neighbouring regions [30].

Here we explore CFR plant-insect diversity relationships using the species rich Restionaceae and their associated insect herbivores as a model system. This is an ideal system for investigating the links between plant and insect diversity since the Restionaceae support a diverse assemblage of insect herbivores, are a dominant component of CFR vegetation and species richness within communities varies substantially across the region [34]. In addition, their fairly uniform growth forms facilitate standardised sampling of herbivore communities. The Restionaceae are wind-pollinated, and thus associated insects are likely interacting antagonistically. Further, our sampling area in the core CFR has low climatic variation, which allows us to assess the plant-insect diversity relationship whilst controlling for broad climatic differences.

First, we ask whether herbivore diversity is correlated with plant species and phylogenetic diversity in the Restionaceae system and use a spatially nested sampling design to explore the strength of this relationship at various spatial scales (i.e. local vs. regional) within the CFR. We predict that if insects are specialised on plants at the species level, we should see a strong relationship between plant and insect richness. If insects are specialised at higher taxonomic levels, plant phylogenetic diversity should be a better predictor of insect richness. It is important to note that, because we sampled a single plant family, and insect herbivores are often specialised at the plant genus or family level [15,16,35], our study is in fact biased against detection of significant plant-insect diversity relationships. Secondly, we ask which aspects of plant diversity (species, phylogenetic, structural or phenophase diversity) best predict insect diversity and whether these relationships are influenced by extrinsic factors (i.e. post-fire vegetation age, and elevation as a proxy for finer scale climatic variance). If plant-insect diversity relationships emerge through evolution of host specialisation, we expect that plant diversity should remain the best predictor of insect diversity when controlling for the influence of environmental gradients and other characteristics of plant communities. Finally, we assemble available datasets to determine how the CFR insect-plant diversity relationship compares to other regions, and to allow preliminary assessment of whether insect diversity in this region follows the same trend as the plants, where diversity is higher than expected from the CFR’s latitude.

## Methods and Materials

### Study system

The African Restionaceae (hereafter restios) is one of the oldest plant clades in the CFR and originated approximately 91.5 million years ago [36]. This wind-pollinated monophyletic clade of reed-like plants contains 350 species [37]; all of which are dioecious and some show dimorphism between male and female reproductive structures. Restio leaves have been reduced to sheaths rolled around the culms at intermittent nodes. While Restionaceae have a typical graminoid growth form, species exhibit differences in plant height, culm diameter and branching of the culms. Restios occur throughout the CFR in habitats that vary in soil type, elevation, groundwater availability, slope, aspect and climate. The high abundance of this group in a variety of habitats makes it ideal for assessing the plant-insect diversity relationship and its correlates in the CFR.

Insect diversity in the CFR has been suggested to be low [32], but more recent studies have found high diversity in galling insects [27] and bees [29]. The sclerophyllous leaves of CFR plants may act as deterrent to folivores [33,38] and the low soil nutrients (leading to low plant nutrients) could favour generalism in herbivorous insects, where insects may switch seasonally between plant species to optimise nutrient intake [19]. Alternately, insects may be specialised on a plant species and only be present in the community when nutrient uptake from that plant species is optimal. Leafhopper species in the tribe Cephalelini (Cicadellidae) have been shown to be specialised on Restionaceae taxa [19,39–41], whereas *Betiscoides* grasshoppers are specialised on the Restionaceae at the family level [42].

### Sampling design

**Fig 1:**
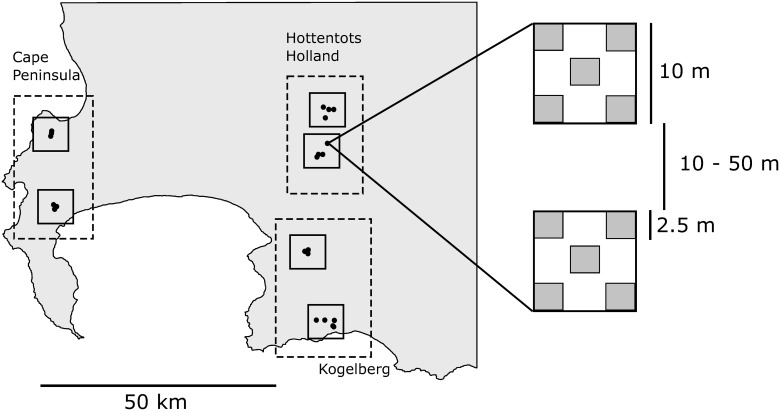
Map depicting sampling area and sampling design. A spatially nested sampling design was employed. Thirty sites (represented by black circles) were sampled twice, once in each season of peak insect activity (autumn and spring). Groups of five sites were spatially aggregated to form six clusters (small grey squares). Two clusters were present in each of the three mountain blocks sampled (large rectangles). Sites consisted of two 10 x 10 m squares situated 10–50 m apart (insert on the right). Each square contained five 2.5 x 2.5 m plots (four corners and centre of the square). These plots were sampled both for Restionaceae plants and all insect herbivores present on Restionaceae plants.

A spatially nested sampling approach was used (Fig 1). Thirty restio-dominated sites were selected for sampling. These were situated on three of the major mountain blocks in the southwestern Cape, namely Hottentots-Holland, Kogelberg and the Cape Peninsula, 50–70 km apart. These mountains experience similar climatic conditions, which allows us to assess the plant-insect diversity relationship whilst controlling for broad climatic differences (see S1 Table). Each mountain block contained two clusters of five sampling sites (15–20 km apart). The five sites in each cluster were situated 100 m to 3 km from one another. Each site consisted of two 10 x 10 m sampling squares located 10 to 50 m apart. Five 2.5 x 2.5 m sampling plots were located within each square (the plots were situated in the corners and centre of each square). Each of the three mountains thus consisted of two clusters, ten sites, twenty squares and a hundred plots. This allowed us to explore the plant-herbivore relationship at various spatial scales, i.e. the plot, square, site, cluster, mountain and regional level. Sites with known restio species composition were chosen to allow us to sample sites that varied in plant species richness independently of elevation and vegetation age. Plots were sampled twice, once during the suggested insect peak season [43] (i.e. Austral spring: August-October 2013) and once six months before this (i.e. Austral autumn: March-April 2013). These sampling periods coincide with the two peaks in Cephalelini abundance [19]. These will hereafter be referred to as spring and autumn respectively.

### Insect sampling and diversity estimation

Insects were collected from all Restionaceae plants occurring in each plot using a modified leaf-blower with a 15 cm diameter nozzle and placed in 70% ethanol. All restios were exhaustively vacuum-sampled for approximately 15–20 seconds per plant and the nozzle was moved systematically up and down the culms. Nwokwu & Sanderson [44] found that using a modified leaf-blower captured more insects than sweep-netting or pitfall trapping, both in terms of richness and abundance. Restios were search-sampled for insects after vacuum sampling to assess its efficiency and also to see whether galling/mining insects were present. Extremely few insects were found by search-sampling and no galling or mining insects were present. Insects were identified to superfamily or family and then sorted into morphospecies. Oliver & Beattie [45] showed morphospecies to be sufficient surrogates for species, especially in estimates of species richness. Samples were matched across seasons. Insect families known to be non-herbivorous were excluded from the dataset. Insect families known to only feed on nectar of plants (absent in restios) were viewed as transient visitors and also excluded from the dataset. No juvenile insects were included in the datset.

The Cephalelini (a dominant insect group in our surveys) were identified to species by dissecting male genitalia and using the species descriptions [39,46], and matching specimens to museum collections (Stellenbosch University, Conservation Ecology and Entomology department). Females were matched to males using external morphology and museum specimens. The insect morphospecies collection is housed in the Botany and Zoology department at Stellenbosch University.

Sampling effectiveness was evaluated by constructing individual based species accumulation curves (number of species found per number of individuals sampled) for the entire region. Accumulation curves (S1 Fig) tended towards saturation and sampling was thus deemed sufficient to allow comparisons between our study and other regions. Rarefaction curves based on the number of plots at each sampling scale showed some variation in the level of approach to saturation between sampling scales (S2–S5 Figs).

Insect alpha diversity was calculated in terms of Hill numbers (or numbers equivalents) of the Shannon diversity index. This diversity metric has the advantage of exhibiting additivity and is not biased towards rare or common species [47]. Alpha diversity was calculated for each plot, square, site, cluster, mountain and the entire region.

### Plant sampling and diversity components

Restionaceae species occurring in each plot were identified using the online interactive key of [48]. Abundances of each restio were recorded for each plot. Plant height was recorded and each plant was placed in a structural height category: 0–0.5 m, 0.5–1 m, 1–1.5 m, 1.5–2 m, and > 2 m. The branching order of plants was recorded as unbranched, branched (each culm branched 1–3 times) or highly branched (each culm branched more than three times). Each plant was placed in a discrete structural group category based on a combination of its height and branching order (e.g. a 0.7 m tall unbranched plant would fall in the “Unbranched_0.5–1 m” bin). The number of plants in flower in each plot was also recorded.

Plant alpha diversity was calculated in the same manner as insect alpha diversity (based on Hill numbers). Plant phylogenetic diversity (PD) was calculated from the Restionaceae phylogeny (Linder & Bouchenak-Khelladi, unpublished) using the R package picante to calculate Faith’s PD. Plant structural diversity was calculated using the alpha diversity metric (Hill numbers) for plant structural groups described above. Structural diversity indicates the number (and abundance) of different structural groups or forms in a plot. A monospecific plot with tall highly branched restios would thus score the same as a monospecific plot of short unbranched restios. However, tall or highly branched restios may provide more feeding niches and support a higher diversity of herbivores. Thus we also used a plant structural complexity metric which aimed to capture the relative amount of culm space available to herbivores in each plot. This was calculated for each plot by multiplying the branching score of each plant (where unbranched plants scored 1, branched plants scored 3 and highly branched plants scored 5) by its height, and summing across all plants in a plot. All of the above calculations were repeated for plots, squares, sites, clusters and mountains.

### Environmental predictors

The elevation and age of the vegetation after the most recent fire was documented for each square (all plots in each square shared the same elevation and vegetation age). The CFR burns regularly (every 10–15 years) and vegetation in squares ranged in post-fire age from 2 to 20 years. Elevation of squares varied between 44 and 968 m above sea level. Elevational gradients often reflect environmental gradients, such as precipitation, temperature, and wind speed differences, and here we use it as a proxy for finer scale climatic differences because available climatic data (i.e. Worldclim) did not have spatial resolution appropriate to our sampling scale and exhibited no significant differences across sampled mountain blocks (S1 Table).

### Data analysis

We first used linear regressions implemented in R [49] to assess the plant-insect diversity relationship in the Restionaceae system independent of other potential predictors (e.g. vegetation structure, vegetation age, environmental variables, etc.). This allowed direct comparison to previous diversity studies [27,28,50]. We tested for relationships between insect species richness and plant species richness and phylogenetic diversity, both locally (i.e. plot, square, site scale) and regionally (i.e. cluster scale). Analysis of variance (ANOVA) was used to assess whether plant and insect species richness in plots, squares and sites varied between mountains. Insect alpha diversity was also regressed onto plant alpha diversity to test whether plant communities with more even abundances host insect communities with even abundances. To test whether an increase in plant richness linearly influenced both insect richness and evenness, we also regressed plant richness onto insect alpha diversity.

Next, general linear models (GLMs) were used to assess the influence of the additional plant diversity components (structural diversity, structural complexity and phenophase diversity) and environmental variables on insect species richness. We conducted five separate GLMs to explore the influence of environmental variables and additional plant diversity components on the insect-plant species richness relationship. We firstly analysed these effects on the, the full dataset (i.e. seasonal data was combined for each square), and then divided the data into subsets using 1) the autumn dataset, 2) the spring dataset, 3) Hemiptera only, 4) Coleoptera only. This allowed us to determine whether diversity relationships differ between seasons and across the dominant insect orders. We used the Akaike information criterion (AIC) from stepwise backward elimination to determine which predictor variables should be included in each of the respective GLMs. Model fit was calculated with all predictors included in the model and predictors were then removed one by one to assess whether model fit improved. The model with the best fit was then used for each of the five GLMs. The function “stepwise” in R [49] was used for this. Response variables (insect species richness) were log_10_(x + 1) transformed where necessary to improve normality. Squares were used as input for all GLMs as this is the sampling scale at which environmental variables were recorded.

To test for differences in the ratio of insect species to plant species between the CFR and other regions, we compiled data from existing studies which assessed the influence of plant diversity on insect diversity. Various combinations of the search terms “insect”, “plant”, “richness”, “relationship”, “diversity”, “Cape Floristic Region” and “tropics” were used to locate studies that relate plant and insect richness on Google Scholar and ISI Web of Science. Studies which 1) only assessed a single taxon of insects, 2) were based on experimental treatments or 3) only sampled a vegetation type (rather than reporting plant species richness) were excluded. Ratios of the total number of insect species sampled to total number of plant species sampled were extracted from the 13 studies we could find that generated datasets comparable to our own (Table 1). As these studies differed in the growth form sampled (tropical studies only sampled trees, while CFR studies sampled shrubs, graminoids and trees) which is likely to strongly influence insect:plant ratios, we first tested for an influence of growth form on ratios using ANOVA. This revealed a strong influence of growth form (F_2,12_ = 11.14, p = 0.001) with tree species housing significantly more insect species than shrubs or graminoids. This is not surprising since [8] showed leaf biomass to be a strong predictor of insect abundance. As a result, trees were excluded from the regional comparison (using Welch’s t-test) of insect:plant ratios from the CFR and other temperate regions (i.e. high plant diversity and low plant diversity temperate areas). We also used a t-test to compare CFR and tropical insect:plant ratios even though region and growth form were entirely confounded in this comparison.

**Table 1:** Number of insect species per plant species for various regions (i.e. the Cape Floristic Region, temperate zones, tropical zones). The number of insect species per plant species is significantly higher in the tropics than the CFR, although this is entirely confounded by plant growth form. The CFR does not have significantly more insect species per plant species than other temperate zones, suggesting regional plant richness and growth form are better predictors of regional insect richness than environmental variables.

Insect:Plant ratio	Growth from	Region	Study
5.82	grass	CFR	This study
1.45	shrub	CFR	Prochess *et al*. (2009) [28]
2.06	shrub	CFR	Pryke & Samways (2008) [43]
4.34	grass	temperate	Zhu *et al*. (2012) [51]
3.24	grass	temperate	Collinge (2000) [52]
13.21	tree	temperate	Novotný *et al*. (2006) [20]
21.60	tree	temperate	Marques *et al*. (2006) [53]
1.24	shrub	temperate	Frenzel & Brandl (2003) [54]
2.18	shrub	temperate	Schaffers *et al*. (2008) [55]
23.27	tree	tropical	Basset & Novotný (1999) [56]
39.86	tree	tropical	Novotný *et al*. (2006) [20]
22.76	tree	tropical	Novotný *et al*. (2012) [50]
20.20	tree	tropical	Leps *et al*. (2001) [57]
35.00	tree	tropical	Novotný & Basset (2000)[58]

## Results

### Plant and insect composition

**Fig 2:**
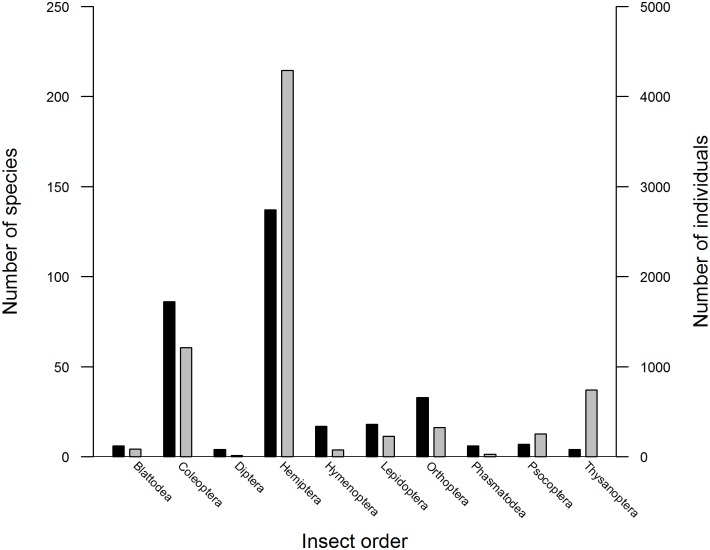
The morphospecies richness and abundance per insect order. Total morphospecies richness (black bars) and abundance (grey bars) for each insect order captured during vacuum sampling surveys of Restionaceae communities across the Cape Floristic Region. Hemiptera dominated herbivore communities on restios. Only herbivorous insects are included. The mean (± SD) insect morphospecies richness for plots was 9.47 ± 4.76 (range: 0–27), for squares 29.25 ± 10.09 (range: 12–60), for sites 44.80 ± 12.80 (range: 26–70), for clusters 126.17 ± 9.06 (range: 118–144) and for mountains 192.67 ± 22.55 (range: 171–216) (Table 2).

A total of 321 herbivorous insect morphospecies were collected (7276 individuals), 221 insect species (3619 individuals) during autumn (March-April 2013), and 195 species (3657 individuals) during spring (August-September 2013). The restio herbivore community was strongly dominated by Hemiptera, both in terms of species richness (42.6% of species) and abundance (58.9% of individuals) (Fig 2). The hemipteran groups, Cephalelini and Fulgoroidea, respectively comprised approximately 10% (773 individuals) and 17% (1237 individuals) of all insect individuals sampled. Although Coleoptera are the largest order of described species globally, they do not dominate this system.

The insects were sampled from 5248 Restionaceae plants (55 species; 11 genera). Mean (± SD) of plant species richness for plots was 3.04 ± 1.80 (range: 1–11), for squares 4.48 ± 2.43 (range: 1–11), for sites 5.57 ± 2.79 (range: 2–14), for clusters 18 ± 7.95 (range: 8–29) and for mountains 28.33 ± 12.50 (range: 16–41) (Table 2).

**Table 2:** The mean insect and plant richness for each sampling scale. The mean (± SD) insect and plant richness of each mountain block (HH: Hottentots-Holland; KB: Kogelberg; CP: Cape Peninsula) is shown for the various sampling scales.

Sampling scale	Group	HH	KB	CP
Plot	Insect	9.62 ± 4.29	10.2 ± 5.64	8.56 ± 4.1
n = 300	Plant	3.02 ± 1.73	3.84 ± 2.11	2.25 ± 1.04
Square	Insect	27.7 ± 7.77	32.6 ± 13.1	27.4 ± 8.13
n = 60	Plant	4.35 ± 2.35	6.15 ± 2.43	2.95 ± 1.23
Site	Insect	42.5 ± 10.3	42.7 ± 13.8	49.2 ± 14.1
n = 30	Plant	5.1 ± 2.28	7.8 ± 2.94	3.8 ± 1.4
Cluster	Insect	124 ± 1.41	121 ± 4.24	134 ± 14.8
n = 6	Plant	17 ± 2.83	27 ± 2.83	10 ± 2.83

### Plant-insect diversity relationship

**Fig 3:**
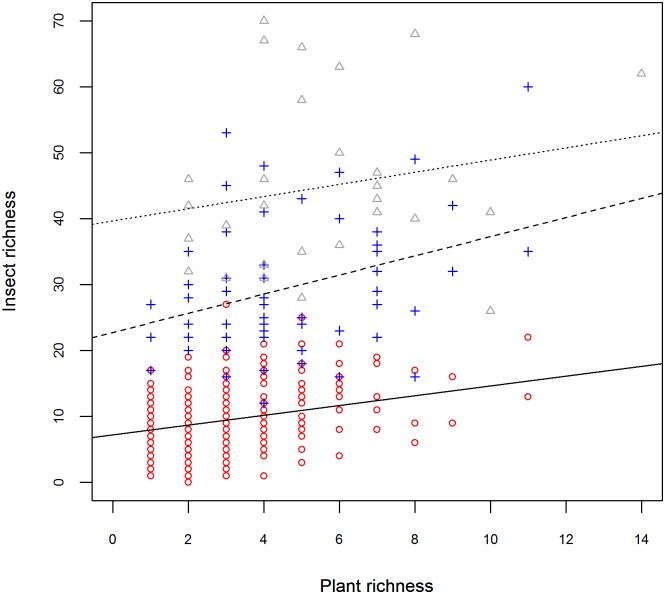
Relationship between plant and insect richness at various sampling scales. The plot scale is represented by a solid line and circles, the square scale is represented by a dashed line and crosses, and the site scale is shown by a dotted line and triangles. The association is positive at the plot and square scales (p < 0.001), but not significant at the site scale.

Insect alpha diversity and species richness were always significantly (or nearly significantly) positively associated with plant diversity (alpha diversity, PD and species richness) at the plot (i.e. 2.5 x 2.5 m sampling unit) and square (10 x 10 m) scales (Table 3). However, plant-insect diversity relationships were not significant at larger sampling scales (Fig 3; Table 3), although the relationship was still positive at the site scale. While significant, plant diversity components only explained a maximum of 11% of variance in insect diversity at the square scale. Plant species richness was a stronger predictor of insect species richness than plant phylogenetic diversity.

**Table 3:** Insect-plant diversity relationship at various spatial sampling scales. While the relationship between insect and plant diversity components was always positive, it was only significant at the smaller sampling scales with the most statistical power, and plant diversity components explained a maximum of 11% of variance in insect diversity.

Scale	Insect species richness ~ Plant species richness		Insect species richness ~ Plant species PD		Insect species α diversity ~ Plant richness		Insect species α diversity ~ Plant species α diversity	
	R^2^	p	R^2^	p	R^2^	p	R^2^	p
Plot (n = 300)	0.076	<0.001	0.040	<0.001	0.052	<0.001	0.031	0.001
Square (n = 60)	0.107	<0.001	0.052	0.043	0.046	0.055	0.049	<0.001
Site (n = 30)	0.006	0.288	-0.020	0.522	0.084	0.065	0.066	0.092
Cluster (n = 6)	0.035	0.338	0.103	0.278	0.047	0.429	0.085	0.292

Plots in the Kogelberg mountain had significantly higher insect species richness than plots on the Cape Peninsula, but no other differences were present (ANOVA: F_2,297_ = 3.231, p = 0.041). No differences were present between mountains for squares (F_2,57_ = 1.751, p = 0.183) or sites (F_2,27_ = 0.880, p = 0.427). Plant species richness per mountain was significantly higher in Kogelberg than in Hottentots Holland, and both of these were higher than the Cape Peninsula for plots (F_2,297_ = 22.19, p < 0.001), squares (F_2,57_ = 11.92, p < 0.001), and sites (F_2,27_ = 9.711, p = 0.002).

For the full dataset, additional variance in insect species richness across squares was explained only by post-fire vegetation age, and not by elevation or plant structural attributes (Table 4). Older vegetation had more insect species. The model including both plant richness and vegetation age explained 16% of variance in insect richness, while plant richness alone explained 11% (Tables 3 and 4). Seasonal patterns differed in that during spring plant structural complexity was the strongest predictor while plant structural diversity and number of plants flowering were stronger predictors in autumn. Similarly, Hemipteran richness was most strongly related to plant structural diversity, while Coleopteran richness was best predicted by plant richness and structural complexity.

**Table 4:** Results of GLM models with insect species richness (at the square level) as the response variable. The model was first run with the entire dataset (i.e. all species for both seasons), and then on subsets of the data (i.e. seasons separately, and main insect orders separately). Data from all 60 sampled squares were included.

Insect richness dataset		Plant components				Extrinsic components		Model fit
		Plant richness	Structural diversity	Structural complexity	Flowering	Vegetation age	Elevation	R^2^
Overall	All	(+) 0.012*				(+) 0.015*		0.16
Subset	Autumn		(+) 0.005*	(-)0.157	(+) 0.004*			0.18
	Spring			(-) 0.008*				0.10
	Hemiptera		(+) 0.022*	(-) 0.486				0.07
	Coleoptera	(+) 0.006*		(-) 0.025*				0.10

P-values of all predictors included in each model are shown and significant values are indicated with an asterisk (*). The last column depicts model fit (R^2^). The direction of effects are indicated in brackets.

### Comparison of insect:plant species ratios between the CFR and other regions

The mean number of insect species per plant species in the CFR did not differ from other temperate regions when the confounding effect of trees was excluded from analysis (t = 0.2350, d.f. = 2.9626, p = 0.83).

Although differences in plant growth form entirely confound the comparison between tropical regions and the CFR, the number of insect species per plant species is significantly lower in the CFR than in the tropics (t = -4.3214, d.f. = 5.753, p = 0.005), despite plant diversity being similar between these regions [59].

## Discussion

The positive relationship between the species richness of Restionaceae plants and their associated herbivorous insect assemblages confirms previous reports of plant-insect diversity linkage in the CFR [27,28,30,43]. Other components of plant diversity (i.e. structural diversity and complexity) and post-fire vegetation age also explained significant amounts of variance in insect species richness and the contribution of these predictors varied between seasons and across the dominant insect orders. However, when combining seasons and insect groups, plant species richness was a persistent significant predictor of insect richness even when accounting for the influence of these other variables.

Insects often specialise on plants at the genus or family level [15,16,35]. Thus the positive diversity relationship we demonstrate within a single plant family is quite remarkable and likely indicates specificity at finer taxonomic levels. This is further supported by plant richness being a better predictor of insect richness than plant phylogenetic diversity. Similar results were found by Kemp et al. [60] where plant-herbivore interactions in the CFR showed high levels of fidelity at the species-level. Our results contrast with Procheş et al. [28] who found plant genera and plant phylogenetic diversity to be the strongest predictors of insect diversity in the CFR, and the taxonomic level at which insects specialise may thus vary between plant groups in the CFR.

The plant-insect diversity relationship was only significant at the smallest sampling scales (< 10 x 10 m). This contrasts with Procheş et al. [28] who found a positive relationship between plant and insect richness up to a 1 km sampling scale in the CFR (in our study a positive, but not significant, trend was present at the site scale—10–50 m). Significance was also absent at the regional sampling scale in Procheş et al. [28]. Based on these results Procheş et al. [28] suggest a direct relationship between plant and insect diversity at fine spatial scales and an indirect association at broader spatial scales where the diversity of these groups rather becomes dependent on abiotic variables, immigration, diversification and extinction rates (also see [61]). However, we cannot exclude the possibility that lack of significance at larger spatial scales reflects reduced statistical power associated with lower sample sizes.

Plant richness continued to positively influence insect richness after accounting for the significant effects of other components of plant diversity and environmental variables. Overall, insect richness increased with both an in increase in plant diversity and an increase in vegetation age, indicating new insect species continuously colonise an area after a fire. Niche diversity may increase up to a point with an increase in vegetation age, allowing for an increase in insect diversity [2,62]. The lack of influence of elevation on restio herbivore richness contrasts what has previously been found in the CFR [43] and globally [63], and suggests that insects associated with the Restionaceae are not strongly influenced by altitudinal environmental gradients, at least across the altitudinal range we sampled (44–968 m).

When only considering seasonal and taxon-based subsets of the data, plant structural complexity and diversity were strong predictors of insect richness, where structurally less complex vegetation had more insect species. This suggests communities with shorter plants, or medium sized unbranched plants, are utilized by more insect species than tall plants. Many of the short plants sampled formed mats that seemed to house high numbers of insects (observation by JEK), where these plants could provide better hiding places from predators [64–66].

Different factors influence Hemiptera and Coleoptera species richness, suggesting different factors could be driving the diversity of different insect orders. These findings align with Koricheva et al. [67] who showed leafhopper, aphid and beetle abundances exhibit different responses to plant richness. Interestingly, different factors are influencing insect richness between seasons and different insect taxa in each season may exhibit different responses to the various plant diversity components.

Despite the significant relationships observed here, limited variance in insect diversity was explained by the models. This is not unique to our study—previousreports on plant-insect diversity linkage also report low R^2^ values. In a meta-analysis Castagenyrol & Jactel [5] found the average R^2^ value to be 0.20 (range: 0.05–0.31). The weak, but significant, plant-insect diversity relationship we detect in our study of herbivores of a single plant family is perhaps not unexpected, as insects are often specialized at the genus or family level, and thus strong diversity relationships are not likely. Further, fine-scale climatic conditions could influence insect distributions, but unfortunately high resolution climatic data were not available to account for this source of variance.

The ratio of insect species to plant species has been suggested to remain constant across latitudes [20]. We found that the ratio of insect species to plant species in the CFR is lower than in the tropics. However, this result likely largely reflects the fact that data available for the CFR- tropical comparison were entirely confounded by growth form (only trees sampled in the tropics and shrubs/grasses in the CFR), and insect:plant species ratios are much higher for trees than for shrubs and grasses. None-the-less, given that the CFR is dominated by shrublands and the tropics by forests, the implication is that regional insect diversity in the CFR is likely much lower than in the tropics, despite similar plant diversity levels, by virtue of the vegetation structure differences alone. In contrast, the ratio of insect to plant species in the CFR is similar to other temperate regions, suggesting that the CFR has high regional insect species richness compared to other temperate systems, due to its exceptional regional plant diversity.

## Conclusion

Our analyses suggest that the association we demonstrate between plant and herbivorous insect diversity in the Restionaceae is unlikely to result primarily from parallel responses of plants and insects to underlying environmental gradients across the CFR. Instead, the fact that the relationship holds across communities with very similar climates, and after accounting for the influence of post-fire vegetation age, elevation, and other aspects of vegetation structure, suggests that it likely arises as a result of specialisation of restio associated insects at taxonomic levels below the plant family level. This mechanism is supported by studies of host-use and preference in restio associated leafhoppers [19,41]. Our results are also similar to those of the handful of other studies in the CFR which have investigated plant-insect diversity linkage, but have sampled across whole plant communities [27,28,30,43], perhaps suggesting that narrow specialisation might be widespread in herbivorous CFR insects. Our approach (i.e. exhaustively sampling relationships from a single plant family) has the advantage of eliminating sampling biases associated with sampling insects from communities of plants with highly divergent plant growth forms and favours sampling of appropriate numbers of plant individuals per species to more accurately estimate diversity of insects associated with them. However, this remains a correlative approach, and experimental assessments of the niche breaths of these insects are required to determine whether these patterns truly result from insect host specialisation. Our comparisons of the CFR insect-plant relationship with the limited set of comparable studies from other areas of the globe suggest that regional insect diversity in the CFR is lower than the tropics, but higher than other temperate regions.

## Supporting Information

S1 Fig Individual-based accumulation curve constructed for (A) autumn and (B) spring. Fitted curves are based on locally weighted scatterplot smoothing (LOESS).
(DOCX)

S2 Fig Rarefaction curves for all squares, based on a combination of the two sampling seasons and using plots as units. (DOCX)

S3 Fig Rarefaction curves for all sites, based on a combination of the two sampling seasons and using plots as units. (DOCX)

S4 Fig Rarefaction curves for all clusters, based on a combination of the two sampling seasons and using plots as units. (DOCX)

S5 Fig Rarefaction curves for (A) the entire region and (B) all mountains, based on a combination of the two sampling seasons and using plots as units. (DOCX)

S1 Table Means and ranges for Bioclim variables are shown for each of the three mountains. Other Bioclim variables showed no variation. The extremely low variation within and between mountains suggest that broad climatic factors are similar between mountains.
(DOCX)
