# Supplementary material for: Significant Local-Scale Plant-Insect Species Richness Relationship Independent of Abiotic Effects in the Temperate Cape Floristic Region Biodiversity Hotspot
Source: PLoS One. 2017 Jan 11;12(1):e0168033. doi: 10.1371/journal.pone.0168033 (PMC5226791; doi:10.1371/journal.pone.0168033)
Supplement: S1 Fig — Fitted curves are based on locally weighted scatterplot smoothing (LOESS). (DOCX) [file pone.0168033.s001.docx]

*Supporting Information*


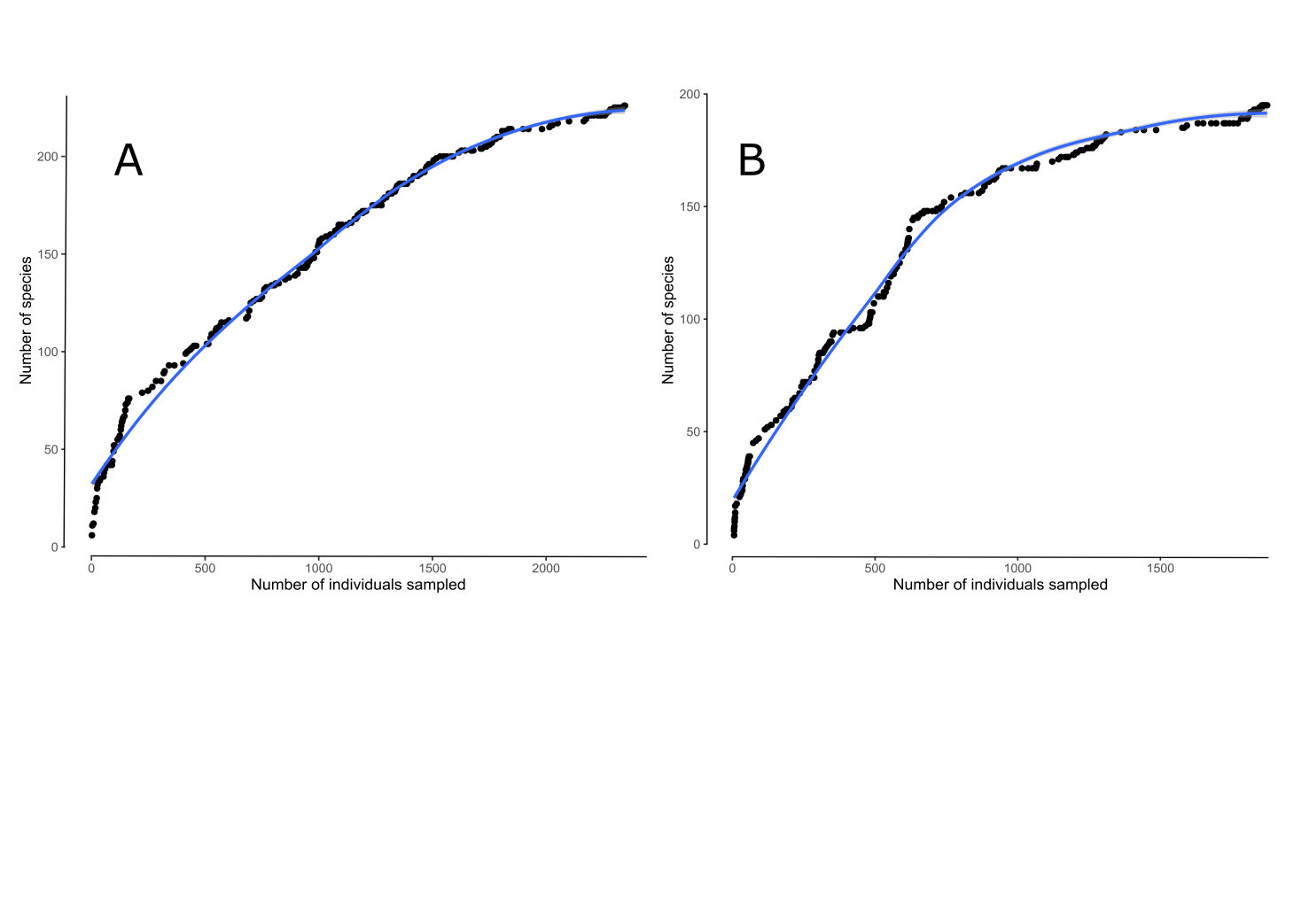


**Figure S1** Individual-based accumulation curve constructed for (A) autumn and (B) spring. Fitted curves are based on locally weighted scatterplot smoothing (LOESS).
