# Supplementary material for: Significant Local-Scale Plant-Insect Species Richness Relationship Independent of Abiotic Effects in the Temperate Cape Floristic Region Biodiversity Hotspot
Source: PLoS One. 2017 Jan 11;12(1):e0168033. doi: 10.1371/journal.pone.0168033 (PMC5226791; doi:10.1371/journal.pone.0168033)
Supplement: S4 Fig — (DOCX) [file pone.0168033.s004.docx]

*Supporting Information*


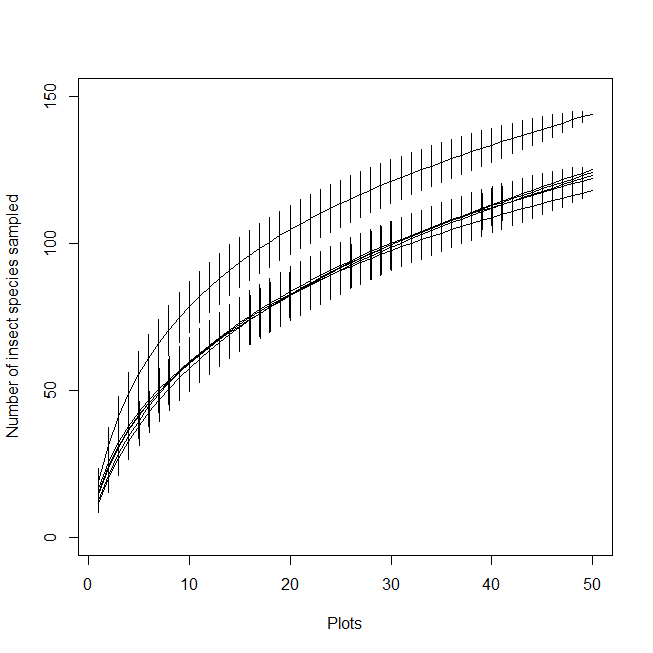


**Figure S4** Rarefaction curves for all clusters, based on a combination of the two sampling seasons and using plots as units.
