# Supplementary material for: Significant Local-Scale Plant-Insect Species Richness Relationship Independent of Abiotic Effects in the Temperate Cape Floristic Region Biodiversity Hotspot
Source: PLoS One. 2017 Jan 11;12(1):e0168033. doi: 10.1371/journal.pone.0168033 (PMC5226791; doi:10.1371/journal.pone.0168033)
Supplement: S1 Table — Other Bioclim variables showed no variation. The extremely low variation within and between mountains suggest that broad climatic factors are similar between mountains. (DOCX) [file pone.0168033.s006.docx]

*Supporting Information*

**Table S1** Means and ranges for Bioclim variables are shown for each of the three mountains. Other Bioclim variables showed no variation. The extremely low variation within and between mountains suggest that broad climatic factors are similar between mountains.

|  | Annual mean temp | Annual precipitation | Annual temperature range | Max temperature of warmest month | Min temperature of coldest month | Precipitation of wettest month |
| --- | --- | --- | --- | --- | --- | --- |
| **Hottentots Holland** | |  |  |  |  |  |
| Mean | 13.59 | 1095.5 | 20.56 | 25.37 | 4.755 | 142.3 |
| Minimum | 12.25 | 1095.5 | 17.35 | 22.1 | 3.6 | 127 |
| Maximum | 15.6 | 1095.5 | 22.7 | 27.55 | 7.45 | 203.5 |
| **Kogelberg** | |  |  |  |  |  |
| Mean | 12.25 | 764 | 17.35 | 22.1 | 7.45 | 127 |
| Minimum | 12.25 | 432.5 | 17.35 | 22.1 | 7.45 | 127 |
| Maximum | 12.25 | 1095.5 | 17.35 | 22.1 | 7.45 | 127 |
| **Cape Peninsula** | |  |  |  |  |  |
| Mean | 15.265 | 1095.5 | 17.35 | 24.28 | 7.45 | 149.95 |
| Minimum | 12.25 | 1095.5 | 17.35 | 22.1 | 7.45 | 127 |
| Maximum | 15.6 | 1095.5 | 17.35 | 27.55 | 7.45 | 203.5 |
